# Supplementary material for: Development, implementation and validation of resource-stratified guidelines in low-income and middle-income countries: a scoping review protocol
Source: BMJ Open. 2022 Sep 28;12(9):e059603. doi: 10.1136/bmjopen-2021-059603 (PMC9528583; doi:10.1136/bmjopen-2021-059603)
Supplement: Supplementary data [file bmjopen-2021-059603supp001.pdf]

Appendices

Appendix I: Search strategy

PubMed Search	Query	Records Retrieved
#1	(resource and(stratified or limited or limitations or stratification or low or constrain or constrained or under or driven or responsible or scarce or targeted or allocate* or management or specific or modified or adapt or adapted or adaptation*))	448,459
#2	("DEVELOPING COUNTRIES"[MH] OR "developing nation" OR "developing country" or "developing countries" OR((LMIC OR LMICS OR LICs OR MICs) OR "DEVELOPING COUNTRIES/ECONOMICS"[MH] OR("Low and middle-income country" OR "lower middle income country" or "Low-and middle-income countries" OR "lower middle-income countries") OR "low-income countries" or "low income country" OR "resource-poor setting" or "transitional country" or "transitional countries"))	175,829
#3	("CLINICAL PRACTICE GUIDELINE" OR "EVIDENCE-BASED RECOMMENDATIONS" OR(GUIDELINE OR GUIDELINES) OR(PROTOCOL) OR "EVIDENCE-BASED MEDICINE"[MH] OR "PRACTICE GUIDELINES AS TOPIC"[MH] or "Health Plan Implementation"[MAJR] OR "GUIDELINE ADHERENCE"[MH] OR "PRACTICE GUIDELINES AS TOPIC"[MH])	1,227,106
#4	#1 AND #2 AND #3	2,796
Filters	Human species; English Language	2,057

Scopus Search	Query	Records Retrieved
#1	TITLE-ABS-KEY (resource* W/3 (stratified OR limited OR limitations OR stratification OR low OR constrain* OR under OR driven OR responsible OR scarce OR targeted OR allocate* OR management OR specific OR modified OR adapt*))	381,768
#2	TITLE-ABS-KEY (("developing countr*" OR "developing nation" OR (lmic* OR lics OR mics) OR "Low* and middle-income countr*" OR ("low-income countr*" OR "resource-poor setting" OR "transitional countr*")))	349,488
#3	TITLE-ABS-KEY ("Clinical Practice Guideline*" OR "Evidence-Based Recommendation*" OR guideline* OR protocol OR "Evidence-Based Medicine" OR "Health Plan Implementation" OR "Guideline Adherence")	2,240,877
#4	#1 AND #2 AND #3	2,168
Filters	Human species; English Language	1,703

Search conducted 4 Nov 2021

Appendix II: Data extraction instrument

Title	Author	Population Characteristics of Developers	Intended Country for Implementation	Rationale for Development	Methodology of Development	Development	Implementation	Validation	Summary of finding	Medical speciality	Practice implications	Recommendations for further development
