# Supplementary material for: Development, implementation and validation of resource-stratified guidelines in low-income and middle-income countries: a scoping review protocol
Source: BMJ Open. 2022 Sep 28;12(9):e059603. doi: 10.1136/bmjopen-2021-059603 (PMC9528583; doi:10.1136/bmjopen-2021-059603)
Supplement: Author's manuscript [file bmjopen-2021-059603.draft_revisions.pdf]

BMJ Open is committed to open peer review. As part of this commitment we make the peer review history of every article we publish publicly available.

When an article is published we post the peer reviewers' comments and the authors' responses online. We also post the versions of the paper that were used during peer review. These are the versions that the peer review comments apply to.

The versions of the paper that follow are the versions that were submitted during the peer review process. They are not the versions of record or the final published versions. They should not be cited or distributed as the published version of this manuscript.

BMJ Open is an open access journal and the full, final, typeset and author-corrected version of record of the manuscript is available on our site with no access controls, subscription charges or pay-per-view fees (http://bmjopen.bmj.com).

If you have any questions on BMJ Open's open peer review process please email info.bmjopen@bmj.com

BMJ Open

Development, Implementation, and Validation of Resource Stratified Guidelines in Low-and Middle-Income Countries: A Scoping Review Protocol

Journal:	*BMJ Open*
Manuscript ID	bmjopen-2021-059603
Article Type:	Protocol
Date Submitted by the Author:	25-Nov-2021

Complete List of Authors:	Griswold, Dylan; Cambridge University, Clinical Neurosciences<br>Venturini, Sara; University of Cambridge, NIHR Global Health Research Group on Neurotrauma; University of Cambridge, Department of Clinical Neurosciences<br>Carney, Nancy; Oregon Health & Science University, Department of Medical Informatics and Clinical Epidemiology<br>Rubiano, Andres M; Universidad El Bosque, Neurosciences Institute; University of Cambridge, NIHR Global Health Research Group on Neurotrauma<br>Hutchinson, Peter; University of Cambridge, NIHR Global Health Research Group on Neurotrauma; University of Cambridge, Department of Clinical Neurosciences<br>Kolias, Angelos; Addenbrooke's Hospital, NIHR Global Health Research Group on Neurotrauma; University of Cambridge, Department of Clinical Neurosciences

Keywords:	Protocols & guidelines < HEALTH SERVICES ADMINISTRATION & MANAGEMENT, Quality in health care < HEALTH SERVICES ADMINISTRATION & MANAGEMENT, International health services < HEALTH SERVICES ADMINISTRATION & MANAGEMENT

SCHOLARONE™
Manuscripts

Development, Implementation, and Validation of Resource Stratified Guidelines in Low-and Middle-Income Countries: A Scoping Review Protocol

Dylan P Griswold[1,2,3] Sara Venturini[1,2] Nancy A. Carney[4] Andres M. Rubiano[1,5,6] Peter J. Hutchinson[1,2] Angelos G. Kolias[1,2]

[1] NIHR Global Health Research Group on Neurotrauma, University of Cambridge, Cambridge, UK [2] Division of Neurosurgery, Department of Clinical Neurosciences, Addenbrooke's Hospital & University of Cambridge, Cambridge, UK [3] Stanford University School of Medicine, Stanford, CA, USA [4] Department of Medical Informatics and Clinical Epidemiology, School of Medicine, Oregon Health & Science University, Portland, Oregon, USA [5] Neuroscience Institute, INUB-MEDITECH Research Group, El Bosque University, Bogotá, Colombia [6] Neurological Surgery Service, Vallesalud Clinic, Cali, Colombia

Keywords: scoping review; developing countries; evidence-based guidelines; resource stratified guidelines; LMICs; low-and middle-income countries; global health

Word count: 1814

Correspondence:

Dylan Griswold

Division of Neurosurgery

Department of Clinical Neurosciences

Addenbrooke's Hospital

University of Cambridge

Cambridge CB2 0QQ

dylgris@gmail.com

ABSTRACT

Introduction: Nearly every field of medicine has some form of clinical practice guidelines. However, only within the past five to ten years has the medical community acknowledged the need for well-developed guidelines tailored to the local healthcare needs and the resources available. In most low-and middle-income countries (LMICs) healthcare workers depend on guidelines developed in high-income countries (HICs), yet many interventions validated in a HIC are ineffective when implemented in an LMIC. The variation in infrastructure, medical personnel, technology, and environmental conditions exhibited in LMICs relative to HICs necessitates a careful appraisal of the evidence base used in clinical guideline recommendations. This review aims to map the use of resource-stratified guidelines across all fields of medicine. The review seeks to answer three questions for the identified guidelines, 1) what was the method of development, 2) have they been implemented, and if so, 3) have they been validated.

Methods

This scoping review will be conducted in accordance with the JBI methodology for scoping reviews. Study selection will be performed by two authors in a two-phase process; if any disagreement arises, a third author will be consulted to make a final decision. Data to be extracted from each study will include, but not be limited to, population characteristics of both developers and intended implementation population, medical specialty, validation status, method of guideline development, whether the study is consensus or evidence-based in addition to a summary of recommendations for practice. Citation screening, full-text review, risk of bias assessment, and extraction of study characteristics and outcomes will be carried out using a web-based software platform that streamlines the production of systematic reviews.

Ethics and dissemination Ethical approval is not required for this review. The plan for dissemination is to publish review findings in a peer-reviewed journal and present findings at high-level conferences that engage the most pertinent stakeholders.

For peer review only

ARTICLE SUMMARY

Strengths and Limitations of this study

- To the best of our knowledge this protocol provides a detailed description of the first scoping review on the development, implementation, and validation of resource-stratified guidelines in low-and middle-income countries.
- The protocol adheres to the rigorous JBI methodology for scoping reviews
- The protocol is being conducted by a multidisciplinary team with extensive experience of conducting high-quality reviews
- Given the narrow specificity of the review, very few papers may be included in the final analysis

For peer review only

INTRODUCTION

Best-practice, evidence-based, clinical practice guidelines have become the gold standard for patient care. However, these guidelines are, by and large, developed by High-Income Countries (HICs). Thus, data extracted from clinical trials and adequate statistically controlled comparative studies used to develop the guidelines are based on the enrolment population. Of the 173,532 trials registered on the International Clinical Trials Registry Platform (ICTRP) between 2005 to 2013, 85% were registered to recruit in HICs.[1] For the same period, 4.7% were registered to recruit in lower-middle-income countries (LMICs) and 0.8% in low-income countries (LICs). Thus, 85% of those studies registered for recruitment in high-income countries have a sample population with biological homogeneity relative to the same study carried out with a sample population enrolled from LMICs with social, cultural, genetic, and infrastructure differences.[1] This difference is real and has implications for implementing guidelines developed for and by HICs in an LMIC. In most LMICs healthcare workers depend on guidelines developed in HICs, yet many interventions validated in a HIC are ineffective when implemented in an LMIC.[2,3] The variation in infrastructure, medical personnel, technology, and environmental conditions exhibited in LMICs relative to HICs necessitates a careful appraisal of the evidence base used in clinical guideline recommendations.

Nearly every field of medicine has some form of clinical practice guidelines.[4] Nevertheless, only within the last 5-10 years has the literature acknowledged the need for well-developed guidelines tailored to the local healthcare needs and the resources available. The WHO has developed guidelines for different topics ranging from antimicrobial resistance and neglected tropical disease to environmental health and health systems.[5] Dr. Charles Mock et al. were renegades in this regard, publishing Trauma Mortality Patterns in Three Nations at Different Economic Levels: Implications for Global Trauma System Development – in 1998.[6] The outcome of the study showed that in all three cities, the majority of deaths occurred in the prehospital setting and that improving emergency care is especially vital in middle-income nations that have already established essential emergency medical services (EMS). Mock then led an initiative between the International Association for the Surgery of Trauma and Surgical Intensive Care (IATSIC) and the WHO to establish the Working Group for Essential Trauma Care. The group sought to formulate a plan to address the difficulties that confront trauma care in LMICs. In 2004, the group published the document, Guidelines for essential trauma care, which set forth a list of essential trauma services in 14 categories of trauma care that the group felt was achievable in nearly every worldwide setting and included the human and physical resources that would ensure their provision.[7]

Though guidelines like those developed by Mock's team are available, they are situational, for which there are no comprehensive guidelines for each of the 14 categories of trauma care.

WHO's current trauma guidelines recognize that some protocols developed by HICs require the most costly resources in the "modern therapeutic armamentarium," and that, "it is unlikely that low- or even middle-income countries will be able to meet these guidelines fully."[7] Thus, the WHO's guidelines list the most essential diagnostic and therapeutic capabilities from HIC trauma guidelines. These recommendations outline essential procedures that the vast majority of LMIC facilities can perform. However, LMICs cannot implement these as official trauma guidelines as they fail to provide recommendations stratified by the resources available across multiple domains and levels of care for severe trauma.

One model that has demonstrated success is the resource-stratified guidelines developed for oncology care in areas with varying levels of resource availability. The Breast Health Global Initiative (BHGI) was organized in 2002 to improve the care of women with breast cancer in low-resource settings.[8] Their initiative was successful in developing clinical practice guidelines that account for varying levels of resources available in differing regions. Their methodology provides a framework for diagnosis and treatment recommendations across four resource levels: basic, limited, enhanced, and maximal.[9–13] In recent years the National Comprehensive Cancer Network (NCCN) have taken their evidence-based, consensus-driven clinical practice guidelines (NCCN Guidelines) developed for high-resouce settings, and have developed a framework for resource-stratifying the guidelines for implementation in resource-limited settings. They have formally designated this framework as the NCCN Framework for Resource Stratification of the NCCN Guidelines (NCCN Framework).[14] Like the BHGI guidelines, the NCCN Framework also identifies four resource environments: basic resources, core resources, enhanced resources, and NCCN Guideline assumed resources. The current library of guidelines includes 16 different types of cancers in addition to breast cancer screening, adult cancer pain, and palliative care.[15]

For peer review only - http://bmjopen.bmj.com/site/about/guidelines.xhtml

According to 2016 data, 47% of the 754,000 verified users of the internet-based guidelines were from 198 countries outside the United States.[14]

While the NCCN Framework has led to the development of a variety of cancer-specific resource-stratified guidelines, they have yet to be translated across other fields of medicine. It is largely unknown if and to what degree other fields of medicine have similar frameworks. Understanding how other fields of medicine have developed and implemented resource-stratified guidelines can inform development of new resource-stratified guidelines for specialities without them. Therefore, this review aims to map the use of resource-stratified guidelines across all fields of medicine. A preliminary search of PROSPERO, MEDLINE, the Cochrane Database of Systematic Reviews, and the JBI Database of Systematic Reviews and Implementation Reports was conducted, and no current or underway systematic reviews on the topic were identified.

The findings of this review will inform stakeholders about relevant models to develop, implement, and evaluate resource-stratified- guidelines in low-resource settings.

METHODS

The proposed scoping review will be conducted in accordance with the JBI methodology for scoping reviews.[16]

Review question

Question: Of the resource-stratified guidelines being used in medicine, what are the methods of development, have they been successfully implemented, and if so, have they been validated? Validation refers to whether the performance of the guidelines was assessed, and if so, the results from the assessment.

Objective: To identify resource-stratified guidelines being used in medicine and assess their methods of development, implementation, and validation.

Patient and public involvement

Patients and the public were not involved in the design of this scoping review protocol.

Data source and search strategy

The search strategy will aim to locate both published and unpublished studies. An initial limited search of PubMed and Scopus was undertaken to identify articles on the topic. The text words contained in the titles and abstracts of relevant articles, and the index terms used to describe the articles were used to develop a full search strategy for PubMed and Scopus. A full search strategy for PubMed and Scopus is detailed in Appendix I. In the second phase of the search, a final search strategy will be adopted for each information source. The reference lists of all selected studies will be screened for additional studies during the third phase of the search.

Selection of studies

Following the search, all identified citations will be collated and uploaded into EndNoteX9 (Clarivate Analytics, PA, USA). The citations will then be imported into Covidence online software (Veritas Health Innovation, Melbourne, Australia) for screening. Two independent researchers will examine titles and abstracts for inclusion. The full text of selected studies will be retrieved and assessed. Full-text studies that do not meet the inclusion criteria will be excluded, and the reasons for exclusion will be provided in an appendix in the final scoping review. Any disagreements that arise between the researchers during either title and abstract screening or full-text screening will be resolved through discussion, or with a third reviewer. The results of the search will be reported in full in the final article and presented in a Preferred Reporting Items for Systematic Reviews and Meta-Analyses extension for Scoping Reviews (PRISMA-ScR) checklist.

For peer review only

For peer review only - http://bmjopen.bmj.com/site/about/guidelines.xhtml

Eligibility criteria

Inclusion criteria

Participants

This scoping review will consider guidelines that were developed for low-and middle-income countries, as defined by world bank data for medical specialties recognized by the European Union and European Economic Area.

Concept

The concept of interest for the proposed scoping review is resource-stratified guidelines developed for low-and middle-income countries across all fields of medicine. This will include, but not be limited to, population, medical specialty, intervention, validation status, method of guideline development, whether the study is consensus or evidence-based in addition to the comparator and outcome measures.

Context

The review will be limited to guidelines developed for low-and middle-income countries, as defined by world bank data for medical specialties recognized by the European Union and European Economic Area.

Types of studies

This scoping review will consider both experimental and quasi-experimental study designs including randomized controlled trials, non-randomized controlled trials, before and after studies, and interrupted time-series studies. In addition, analytical observational studies, including prospective and retrospective cohort studies, case-control studies, and analytical cross-sectional studies, will be considered for inclusion.

Studies published in English and English abstracts of foreign language studies will be included. All studies will be considered regardless of publication date.

The PCC inclusion criteria is summarized in Table 1.

Data extraction

Data will be extracted from the papers included in the review by two independent researchers using the data extraction instrument (Appendix II). The following information will be extracted from the articles: (1) study title; (2) author; (3) population characteristics of guideline developers; (4) intended population for implementation; (5) rationale for development; (6) methodology (7) development; (8) implementation; (9) validation; (10) summary of findings; (11) medical speciality; 12) practice implications; (13) recommendations for further development

The draft data extraction tool will be modified and revised as necessary during the process of extracting data from each included study. Modifications will be detailed in the full scoping review report. Any disagreements that arise between the reviewers will be resolved through discussion, or with a third reviewer. Authors of papers will be contacted to request missing or additional data, where required.

Data presentation

The extracted data will be presented in tabular form and as a narrative summary that aligns with the aim of this scoping review. The table will report: i) distribution of studies by countries of guideline origin and intended population; ii) methodology of development; iii) implementation and validation; iv); summary of recommendations. Graphical representations may be used, including bar charts, line charts, pie charts, and diagrams.

For peer review only

Ethics and dissemination

No ethical approval will be required, as this review is based on already published data and does not involve interaction with human subjects. The plan for dissemination, however, is to publish the findings of the review in a peer-reviewed journal and present findings at high- level international conferences that engage the most pertinent stakeholders.

DISCUSSION

This protocol has been rigorously developed and designed specifically to determine the methods of development, implementation, and validation of resource-stratified guidelines being used across all fields of medicine. The findings of this review will inform stakeholders about relevant models to develop, implement, and evaluate resource-stratified guidelines in low-resource settings.

Limitations

This study will be limited by the relative novelty of guideline development for LMICs. Based upon our initial search there are very few examples of resource-stratified guidelines. However, the purpose of the review is to identify those that do exist and to extract the elements that will aid in future development of resource-stratified guidelines in LMICs.

For peer review only

Contributors All authors contributed to the methodology and writing of this review.

Funding This work is supported by the NIHR Global Health Research Group on Neurotrauma, which was commissioned by the National Institute for Health Research (NIHR) using UK aid from the UK Government (project 16/137/105). The views expressed in this publication are those of the author(s) and not necessarily those of the NIHR or the Department of Health and Social Care. D.P.G. is supported by the Gates Cambridge Trust (OPP1144). P.J.H. is supported by a Research Professorship from the NIHR, the NIHR Cambridge Biomedical Research Centre, a European Union Seventh Framework Program grant (CENTER-TBI; grant no. 602,150), and the Royal College of Surgeons of England. A.G.K. is supported by a Clinical Lectureship, School of Clinical Medicine, University of Cambridge and the Royal College of Surgeons of England.

Competing interests None declared.

Patient consent for publication Not required.

Data statement: This review is based on previously published data. Any relevant data will be published with the review as either an appendix or as an online supplement.

Acknowledgements The authors would like to thank Isla Kuhn, Head of Medical Library Services, University of Cambridge Medical Library for her assistance in developing the search strategy

Open access This is an open access article distributed in accordance with the Creative Commons Attribution Non Commercial (CC BY-NC 4.0) license, which permits others to distribute, remix, adapt, build upon this work non-commercially, and license their derivative works on different terms, provided the original work is properly cited, appropriate credit is given, any changes made indicated, and the use is non-commercial. See: http://creativecommons.org/licenses/by-nc/4.0/.

License to BMJ Group for Publication: I *Dylan Griswold,* the Corresponding Author of this article contained within the original manuscript which includes any diagrams & photographs within and any related or stand-alone film submitted (the Contribution") has the right to grant on behalf of all authors and does grant on behalf of all authors, a licence to the BMJ Publishing Group Ltd and its licencees, to permit this Contribution (if accepted) to be published in the BMJ and any other BMJ Group products and to exploit all subsidiary rights, as set out in our licence set out at: http://www.bmj.com/about-bmj/resources-authors/forms-policies-and-checklists/copyright-open-access-and-permission-reuse.

For peer review only

REFERENCES

1 Viergever RF, Li K. Trends in global clinical trial registration: an analysis of numbers of registered clinical trials in different parts of the world from 2004 to 2013. *Bmj Open* 2015;5:e008932. doi:10.1136/bmjopen-2015-008932

2 Buekens P, Keusch G, Belizan J, *et al.* Evidence-Based Global Health. *Jama* 2004;291:2639–41. doi:10.1001/jama.291.21.2639

3 Olayemi E, Asare EV, Kuma AAB. Guidelines in lower-middle income countries. *Brit J Haematol* 2017;177:846–54. doi:10.1111/bjh.14583

4 WEISZ G, CAMBROSIO A, KEATING P, *et al.* The Emergence of Clinical Practice Guidelines: The Emergence of Clinical Practice Guidelines. *Milbank Q* 2007;85:691–727. doi:10.1111/j.1468-0009.2007.00505.x

5 Organization"] ["World Health. WHO guidelines approved by the Guidelines Review Committee. 2020.

6 Mock CN, Jurkovich GJ, nii-Amon-Kotei D, *et al.* Trauma Mortality Patterns in Three Nations at Different Economic Levels: Implications for Global Trauma System Development. *J Trauma Inj Infect Critical Care* 1998;44:804–14. doi:10.1097/00005373-199805000-00011

7 Mock C, Organization) Essential Trauma Care Project (World Health, Organization. World Health, *et al. Guidelines for essential trauma care*. Geneva: : World Health Organization 2004.

8 Anderson BO, Braun S, Carlson RW, *et al.* Overview of Breast Health Care Guidelines for Countries with Limited Resources. *Breast J* 2003;9:S42–50. doi:10.1046/j.1524-4741.9.s2.3.x

9 Anderson BO, Carlson RW. Guidelines for Improving Breast Health Care in Limited Resource Countries: The Breast Health Global Initiative. *J Natl Compr Canc Ne* 2007;5:348–55. doi:10.6004/jnccn.2007.0031

10 Anderson BO, Yip C, Smith RA, *et al.* Guideline implementation for breast healthcare in low-income and middle-income countries. *Cancer* 2008;113:2221–43. doi:10.1002/cncr.23844

11 Anderson BO, Shyyan R, Eniu A, *et al.* Breast Cancer in Limited-Resource Countries: An Overview of the Breast Health Global Initiative 2005 Guidelines. *Breast J* 2006;12:S3–15. doi:10.1111/j.1075-122x.2006.00199.x

12 Anderson BO. Breast healthcare and cancer control in limited-resource countries: a framework for change. *Nat Clin Pract Oncol* 2006;3:4–5. doi:10.1038/ncponc0397

13 Anderson BO, Yip C-H, Ramsey SD, *et al.* Breast Cancer in Limited-Resource Countries: Health Care Systems and Public Policy. *Breast J* 2006;12:S54–69. doi:10.1111/j.1075-122x.2006.00203.x

14 Carlson RW, Scavone JL, Koh W-J, *et al.* NCCN Framework for Resource Stratification: A Framework for Providing and Improving Global Quality Oncology Care. *J National Compr Cancer Netw Jnccn* 2016;14:961–9. doi:10.6004/jnccn.2016.0103

For peer review only

15 Network NCC. NCCN Framework for Resource Stratification of NCCN Guidelines (NCCN Framework™). 2020.https://www.nccn.org/framework/ (accessed 29 Dec 2020).

16 Peters MD, Godfrey CM, Khalil H, *et al.* Guidance for conducting systematic scoping reviews. *Int J Evid Based Healthc* 2015;13:141–6. doi:10.1097/xeb.0000000000000050

Participants	Guidelines developed for low-and middle-income countries across all fields of medicine
Concept	Development, implementation, and validation of resource-stratfied guidelines
Context	Limited to guidelines developed for low-and middle-income countries, as defined by world bank data for medical specialties recognized by the European Union and European Economic Area

Table 1. PCC inclusion criteria

For peer review only

Appendices

Appendix I: Search strategy

PubMed Search	Query	Records Retrieved
#1	(resource and(stratified or limited or limitations or stratification or low or constrain or constrained or under or driven or responsible or scarce or targeted or allocate* or management or specific or modified or adapt or adapted or adaptation*))	448,459
#2	("DEVELOPING COUNTRIES"[MH] OR "developing nation" OR "developing country" or "developing countries" OR((LMIC OR LMICS OR LICs OR MICs) OR "DEVELOPING COUNTRIES/ECONOMICS"[MH] OR("Low and middle-income country" OR "lower middle income country" or "Low-and middle-income countries" OR "lower middle-income countries") OR "low-income countries" or "low income country" OR "resource-poor setting" or "transitional country" or "transitional countries"))	175,829
#3	("CLINICAL PRACTICE GUIDELINE" OR "EVIDENCE-BASED RECOMMENDATIONS" OR(GUIDELINE OR GUIDELINES) OR(PROTOCOL) OR "EVIDENCE-BASED MEDICINE"[MH] OR "PRACTICE GUIDELINES AS TOPIC"[MH] or "Health Plan Implementation"[MAJR] OR "GUIDELINE ADHERENCE"[MH] OR "PRACTICE GUIDELINES AS TOPIC"[MH])	1,227,106
#4	#1 AND #2 AND #3	2,796
Filters	Human species; English Language	2,057

Scopus Search	Query	Records Retrieved
#1	TITLE-ABS-KEY (resource* W/3 (stratified OR limited OR limitations OR stratification OR low OR constrain* OR under OR driven OR responsible OR scarce OR targeted OR allocate* OR management OR specific OR modified OR adapt*))	381,768
#2	TITLE-ABS-KEY (("developing countr*" OR "developing nation" OR (lmic* OR lics OR mics) OR "Low* and middle-income countr*" OR ("low-income countr*" OR "resource-poor setting" OR "transitional countr*")))	349,488
#3	TITLE-ABS-KEY ("Clinical Practice Guideline*" OR "Evidence-Based Recommendation*" OR guideline* OR protocol OR "Evidence-Based Medicine" OR "Health Plan Implementation" OR "Guideline Adherence")	2,240,877
#4	#1 AND #2 AND #3	2,168
Filters	Human species; English Language	1,703

Search conducted 4 Nov 2021

Appendix II: Data extraction instrument

Title	Author	Population Characteristics of Developers	Intended Country for Implementation	Rationale for Development	Methodology of Development	Development	Implementation	Validation	Summary of finding	Medical speciality	Practice implications	Recommendations for further development

For peer review only

BMJ Open

Development, Implementation, and Validation of Resource Stratified Guidelines in Low-and Middle-Income Countries: A Scoping Review Protocol

Journal:	*BMJ Open*
Manuscript ID	bmjopen-2021-059603.R1
Article Type:	Protocol
Date Submitted by the Author:	15-Apr-2022
Complete List of Authors:	Griswold, Dylan; Cambridge University, Clinical Neurosciences<br>Venturini, Sara; University of Cambridge, NIHR Global Health Research Group on Neurotrauma; University of Cambridge, Department of Clinical Neurosciences<br>Carney, Nancy; Oregon Health & Science University, Department of Medical Informatics and Clinical Epidemiology<br>Rubiano, Andres M; Universidad El Bosque, Neurosciences Institute; University of Cambridge, NIHR Global Health Research Group on Neurotrauma<br>Hutchinson, Peter; University of Cambridge, NIHR Global Health Research Group on Neurotrauma; University of Cambridge, Department of Clinical Neurosciences<br>Kolias, Angelos; Addenbrooke's Hospital, NIHR Global Health Research Group on Neurotrauma; University of Cambridge, Department of Clinical Neurosciences
**Primary Subject Heading**:	Evidence based practice
Secondary Subject Heading:	Global health, Research methods
Keywords:	Protocols & guidelines < HEALTH SERVICES ADMINISTRATION & MANAGEMENT, Quality in health care < HEALTH SERVICES ADMINISTRATION & MANAGEMENT, International health services < HEALTH SERVICES ADMINISTRATION & MANAGEMENT

SCHOLARONE™
Manuscripts

Development, Implementation, and Validation of Resource Stratified Guidelines in Low-and Middle-Income Countries: A Scoping Review Protocol

Dylan P Griswold[1,2,3] Sara Venturini[1,2] Nancy A. Carney[4] Andres M. Rubiano[1,5,6] Peter J. Hutchinson[1,2] Angelos G. Kolias[1,2]

[1] NIHR Global Health Research Group on Neurotrauma, University of Cambridge, Cambridge, UK [2] Division of Neurosurgery, Department of Clinical Neurosciences, Addenbrooke's Hospital & University of Cambridge, Cambridge, UK [3] Stanford University School of Medicine, Stanford, CA, USA [4] Department of Medical Informatics and Clinical Epidemiology, School of Medicine, Oregon Health & Science University, Portland, Oregon, USA [5] Neuroscience Institute, INUB-MEDITECH Research Group, El Bosque University, Bogotá, Colombia [6] Neurological Surgery Service, Vallesalud Clinic, Cali, Colombia

Keywords: scoping review; developing countries; evidence-based guidelines; resource stratified guidelines; LMICs; low-and middle-income countries; global health

Word count: 1814

Correspondence:

Dylan Griswold

Division of Neurosurgery

Department of Clinical Neurosciences

Addenbrooke's Hospital

University of Cambridge

Cambridge CB2 0QQ

dylgris@gmail.com

ABSTRACT

Introduction: Nearly every field of medicine has some form of clinical practice guidelines. However, only within the past five to ten years has the medical community acknowledged the need for well-developed guidelines tailored to the local healthcare needs and the resources available. In most low-and middle-income countries (LMICs) healthcare workers depend on guidelines developed in high-income countries (HICs), yet many interventions validated in a HIC are ineffective when implemented in an LMIC. The variation in infrastructure, medical personnel, technology, and environmental conditions exhibited in LMICs relative to HICs necessitates a careful appraisal of the evidence base used in clinical guideline recommendations. This review aims to map the use of resource-stratified guidelines across all fields of medicine. The review seeks to answer three questions for the identified guidelines, 1) what was the method of development, 2) have they been implemented, and if so, 3) have they been validated.

Methods

This scoping review will be conducted in accordance with the JBI methodology for scoping reviews. Study selection will be performed by two authors in a two-phase process; if any disagreement arises, a third author will be consulted to make a final decision. Data to be extracted from each study will include, but not be limited to, population characteristics of both developers and intended implementation population, medical specialty, validation status, method of guideline development, whether the study is consensus or evidence-based in addition to a summary of recommendations for practice. Citation screening, full-text review, risk of bias assessment, and extraction of study characteristics and outcomes will be carried out using a web-based software platform that streamlines the production of systematic reviews.

Ethics and dissemination Ethical approval is not required for this review. The plan for dissemination is to publish review findings in a peer-reviewed journal and present findings at high-level conferences that engage the most pertinent stakeholders.

For peer review only

ARTICLE SUMMARY

Strengths and Limitations of this study

- To the best of our knowledge this protocol provides a detailed description of the first scoping review on the development, implementation, and validation of resource-stratified guidelines in low-and middle-income countries.
- The protocol adheres to the rigorous JBI methodology for scoping reviews
- The protocol is being conducted by a multidisciplinary team with extensive experience of conducting high-quality reviews
- Given the narrow specificity of the review, very few papers may be included in the final analysis

For peer review only

INTRODUCTION

Best-practice, evidence-based, clinical practice guidelines have become the gold standard for patient care. However, these guidelines are, by and large, developed by High-Income Countries (HICs). Thus, data extracted from clinical trials and adequate statistically controlled comparative studies used to develop the guidelines are based on the enrolment population. Of the 173,532 trials registered on the International Clinical Trials Registry Platform (ICTRP) between 2005 to 2013, 85% were registered to recruit in HICs.[1] For the same period, 4.7% were registered to recruit in lower-middle-income countries (LMICs) and 0.8% in low-income countries (LICs). Thus, 85% of those studies registered for recruitment in high-income countries have a very different sample population relative to the same study carried out with a sample population enrolled from LMICs with social, cultural, genetic, and infrastructure differences.[1] This difference is real and has implications for implementing guidelines developed for and by HICs in an LMIC. In most LMICs healthcare workers depend on guidelines developed in HICs, yet many interventions validated in a HIC are ineffective when implemented in an LMIC.[2,3] The variation in infrastructure, medical personnel, technology, and environmental conditions exhibited in LMICs relative to HICs necessitates a careful appraisal of the evidence base used in clinical guideline recommendations.

Nearly every field of medicine has some form of clinical practice guidelines.[4] Nevertheless, only within the last 5-10 years has the literature acknowledged the need for well-developed guidelines tailored to the local healthcare needs and the resources available. The WHO has developed guidelines for different topics ranging from antimicrobial resistance and neglected tropical disease to environmental health and health systems.[5] Dr. Charles Mock et al. were renegades in this regard, publishing Trauma Mortality Patterns in Three Nations at Different Economic Levels: Implications for Global Trauma System Development – in 1998.[6] The outcome of the study showed that in all three cities, the majority of deaths occurred in the prehospital setting and that improving emergency care is especially vital in middle-income nations that have already established essential emergency medical services (EMS). Mock then led an initiative between the International Association for the Surgery of Trauma and Surgical Intensive Care (IATSIC) and the WHO to establish the Working Group for Essential Trauma Care. The group sought to formulate a plan to address the difficulties that confront trauma care in LMICs. In 2004, the group published the document, Guidelines for essential trauma care, which set forth a list of essential trauma services in 14 categories of trauma care that the group felt was achievable in nearly every worldwide setting and included the human and physical resources that would ensure their provision.[7]

Though guidelines like those developed by Mock's team are available, they are situational, for which there are no comprehensive guidelines for each of the 14 categories of trauma care.

WHO's current trauma guidelines recognize that some protocols developed by HICs require the most costly resources in the "modern therapeutic armamentarium," and that, "it is unlikely that low- or even middle-income countries will be able to meet these guidelines fully."[7] Thus, the WHO's guidelines list the most essential diagnostic and therapeutic capabilities from HIC trauma guidelines. These recommendations outline essential procedures that the vast majority of LMIC facilities can perform. However, LMICs cannot implement these as official trauma guidelines as they fail to provide recommendations stratified by the resources available across multiple domains and levels of care for severe trauma.

One model that has demonstrated success is the resource-stratified guidelines developed for oncology care in areas with varying levels of resource availability. The Breast Health Global Initiative (BHGI) was organized in 2002 to improve the care of women with breast cancer in low-resource settings.[8] Their initiative was successful in developing clinical practice guidelines that account for varying levels of resources available in differing regions. Their methodology provides a framework for diagnosis and treatment recommendations across four resource levels: basic, limited, enhanced, and maximal.[9–13] In recent years the National Comprehensive Cancer Network (NCCN) have taken their evidence-based, consensus-driven clinical practice guidelines (NCCN Guidelines) developed for high-resouce settings, and have developed a framework for resource-stratifying the guidelines for implementation in resource-limited settings. They have formally designated this framework as the NCCN Framework for Resource Stratification of the NCCN Guidelines (NCCN Framework).[14] Like the BHGI guidelines, the NCCN Framework also identifies four resource environments: basic resources, core resources, enhanced resources, and NCCN Guideline assumed resources. The current library of guidelines includes 16 different types of cancers in addition to breast cancer screening, adult cancer pain, and palliative care.[15]

For peer review only - http://bmjopen.bmj.com/site/about/guidelines.xhtml

According to 2016 data, 47% of the 754,000 verified users of the internet-based guidelines were from 198 countries outside the United States.[14]

While the NCCN Framework, has led to the development of a variety of cancer-specific resource-stratified guidelines, they have yet to be translated across other fields of medicine. The American Society of Clinical Oncology (ASCO) and the Asian Oncology Society have also developed their own set of cancer-specific resource-stratified guidelines.[16,17] It is largely unknown if and to what degree other fields of medicine have similar frameworks. Understanding how other fields of medicine have developed and implemented resource-stratified guidelines can inform development of new resource-stratified guidelines for specialities without them. Therefore, this review aims to map the use of resource-stratified guidelines across all fields of medicine. A preliminary search of PROSPERO, MEDLINE, the Cochrane Database of Systematic Reviews, and the JBI Database of Systematic Reviews and Implementation Reports was conducted, and no current or underway systematic reviews on the topic were identified.

The findings of this review will inform stakeholders about relevant models to develop, implement, and evaluate resource-stratified- guidelines in low-resource settings.

METHODS

The proposed scoping review will be conducted in accordance with the JBI methodology for scoping reviews.[18]

Review question

Question: Of the resource-stratified guidelines being used in medicine, what are the methods of development, have they been successfully implemented, and if so, have they been validated? Validation refers to whether the performance of the guidelines was assessed, and if so, the results from the assessment.

Objective: To identify resource-stratified guidelines being used in medicine and assess their methods of development, implementation, and validation.

Patient and public involvement

Patients and the public were not involved in the design of this scoping review protocol.

Data source and search strategy

The search strategy will aim to locate both published and unpublished studies from inception to November 2021. An initial limited search of PubMed and Scopus was undertaken to identify articles on the topic. The text words contained in the titles and abstracts of relevant articles, and the index terms used to describe the articles were used to develop a full search strategy for PubMed and Scopus. A full search strategy for PubMed and Scopus is detailed in Appendix I. In the second phase of the search, a final search strategy will be adopted for each information source. The reference lists of all selected studies will be screened for additional studies during the third phase of the search. Unpublished studies will be captured through scanning societal meetings for abstracts and consensus-based guideline development conferences.

Selection of studies

Following the search, all identified citations will be collated and uploaded into EndNoteX9 (Clarivate Analytics, PA, USA). The citations will then be imported into Covidence online software (Veritas Health Innovation, Melbourne, Australia) for screening. Two independent researchers will examine titles and abstracts for inclusion. The full text of selected studies will be retrieved and assessed. Full-text studies that do not meet the inclusion criteria will be excluded, and the reasons for exclusion will be provided in an appendix in the final scoping review. Any disagreements that arise between the researchers during either title and abstract screening or full-text screening will be resolved through discussion, or with a third reviewer. The results of the search will be reported in full in the final article and presented in a Preferred Reporting Items for Systematic Reviews and Meta-Analyses extension for Scoping Reviews (PRISMA-ScR) checklist.

For peer review only - http://bmjopen.bmj.com/site/about/guidelines.xhtml

Eligibility criteria

Inclusion criteria

Participants

This scoping review will consider guidelines that were developed for low-and middle-income countries, as defined by world bank data for medical specialties recognized by the European Union and European Economic Area.

Concept

The concept of interest for the proposed scoping review is resource-stratified guidelines developed for low-and middle-income countries across all fields of medicine. This will include, but not be limited to, population, medical specialty, intervention, validation status, method of guideline development, whether the study is consensus or evidence-based in addition to the comparator and outcome measures.

Context

The review will be limited to guidelines developed for low-and middle-income countries, as defined by world bank data for medical specialties recognized by the European Union and European Economic Area.

Types of studies

This scoping review will consider both experimental and quasi-experimental study designs including randomized controlled trials, non-randomized controlled trials, before and after studies, and interrupted time-series studies. In addition, analytical observational studies, including prospective and retrospective cohort studies, case-control studies, and analytical cross-sectional studies, will be considered for inclusion.

Studies published in English and English abstracts of foreign language studies will be included. All studies will be considered regardless of publication date.

The PCC inclusion criteria is summarized in Table 1.

Data extraction

Data will be extracted from the papers included in the review by two independent researchers using the data extraction instrument (Appendix II). The following information will be extracted from the articles: (1) study title; (2) author; (3) population characteristics of guideline developers; (4) intended population for implementation; (5) rationale for development; (6) methodology (7) development; (8) implementation; (9) validation; (10) summary of findings; (11) medical speciality; 12) practice implications; (13) recommendations for further development

The draft data extraction tool will be modified and revised as necessary during the process of extracting data from each included study. Modifications will be detailed in the full scoping review report. Any disagreements that arise between the reviewers will be resolved through discussion, or with a third reviewer. Authors of papers will be contacted to request missing or additional data, where required.

Data presentation

The extracted data will be presented in tabular form and as a narrative summary that aligns with the aim of this scoping review. The table will report: i) distribution of studies by countries of guideline origin and intended population; ii) methodology of development; iii) implementation and validation; iv); summary of recommendations. Graphical representations may be used, including bar charts, line charts, pie charts, and diagrams.

For peer review only

Ethics and dissemination

No ethical approval will be required, as this review is based on already published data and does not involve interaction with human subjects. The plan for dissemination, however, is to publish the findings of the review in a peer-reviewed journal and present findings at high- level international conferences that engage the most pertinent stakeholders.

DISCUSSION

This protocol has been rigorously developed and designed specifically to determine the methods of development, implementation, and validation of resource-stratified guidelines being used across all fields of medicine. The findings of this review will inform stakeholders about relevant models to develop, implement, and evaluate resource-stratified guidelines in low-resource settings.

Limitations

This study will be limited by the relative novelty of guideline development for LMICs. Based upon our initial search there are very few examples of resource-stratified guidelines. However, the purpose of the review is to identify those that do exist and to extract the elements that will aid in future development of resource-stratified guidelines in LMICs.

For peer review only

Contributors DG, AK, PJH, AMR conceived the review. NA and SA helped with the development of the methods. DG drafted the manuscript and all authors contributed to its revision.

Funding This work is supported by the NIHR Global Health Research Group on Neurotrauma, which was commissioned by the National Institute for Health Research (NIHR) using UK aid from the UK Government (project 16/137/105). The views expressed in this publication are those of the author(s) and not necessarily those of the NIHR or the Department of Health and Social Care. D.P.G. is supported by the Gates Cambridge Trust (OPP1144). P.J.H. is supported by a Research Professorship from the NIHR, the NIHR Cambridge Biomedical Research Centre, a European Union Seventh Framework Program grant (CENTER-TBI; grant no. 602,150), and the Royal College of Surgeons of England. A.G.K. is supported by a Clinical Lectureship, School of Clinical Medicine, University of Cambridge and the Royal College of Surgeons of England.

Competing interests None declared.

Patient consent for publication Not required.

Data statement: This review is based on previously published data. Any relevant data will be published with the review as either an appendix or as an online supplement.

Acknowledgements The authors would like to thank Isla Kuhn, Head of Medical Library Services, University of Cambridge Medical Library for her assistance in developing the search strategy

Open access This is an open access article distributed in accordance with the Creative Commons Attribution Non Commercial (CC BY-NC 4.0) license, which permits others to distribute, remix, adapt, build upon this work non-commercially, and license their derivative works on different terms, provided the original work is properly cited, appropriate credit is given, any changes made indicated, and the use is non-commercial. See: http://creativecommons.org/licenses/by-nc/4.0/.

License to BMJ Group for Publication: I *Dylan Griswold,* the Corresponding Author of this article contained within the original manuscript which includes any diagrams & photographs within and any related or stand-alone film submitted (the Contribution") has the right to grant on behalf of all authors and does grant on behalf of all authors, a licence to the BMJ Publishing Group Ltd and its licencees, to permit this Contribution (if accepted) to be published in the BMJ and any other BMJ Group products and to exploit all subsidiary rights, as set out in our licence set out at: http://www.bmj.com/about-bmj/resources-authors/forms-policies-and-checklists/copyright-open-access-and-permission-reuse.

For peer review only

1 Viergever RF, Li K. Trends in global clinical trial registration: an analysis of numbers of registered clinical trials in different parts of the world from 2004 to 2013. *Bmj Open* 2015;**5**:e008932. doi:10.1136/bmjopen-2015-008932

2 Buekens P, Keusch G, Belizan J, *et al.* Evidence-Based Global Health. *Jama* 2004;**291**:2639–41. doi:10.1001/jama.291.21.2639

3 Olayemi E, Asare EV, Kuma AAB. Guidelines in lower-middle income countries. *Brit J Haematol* 2017;**177**:846–54. doi:10.1111/bjh.14583

4 WEISZ G, CAMBROSIO A, KEATING P, *et al.* The Emergence of Clinical Practice Guidelines: The Emergence of Clinical Practice Guidelines. *Milbank Q* 2007;**85**:691–727. doi:10.1111/j.1468-0009.2007.00505.x

5 Organization"] ["World Health. WHO guidelines approved by the Guidelines Review Committee. 2020.

6 Mock CN, Jurkovich GJ, nii-Amon-Kotei D, *et al.* Trauma Mortality Patterns in Three Nations at Different Economic Levels: Implications for Global Trauma System Development. *J Trauma Inj Infect Critical Care* 1998;**44**:804–14. doi:10.1097/00005373-199805000-00011

7 Mock C, Organization) Essential Trauma Care Project (World Health, Organization. World Health, *et al. Guidelines for essential trauma care*. Geneva: : World Health Organization 2004.

8 Anderson BO, Braun S, Carlson RW, *et al.* Overview of Breast Health Care Guidelines for Countries with Limited Resources. *Breast J* 2003;**9**:S42–50. doi:10.1046/j.1524-4741.9.s2.3.x

9 Anderson BO, Carlson RW. Guidelines for Improving Breast Health Care in Limited Resource Countries: The Breast Health Global Initiative. *J Natl Compr Canc Ne* 2007;**5**:348–55. doi:10.6004/jnccn.2007.0031

10 Anderson BO, Yip C, Smith RA, *et al.* Guideline implementation for breast healthcare in low-income and middle-income countries. *Cancer* 2008;**113**:2221–43. doi:10.1002/cncr.23844

11 Anderson BO, Shyyan R, Eniu A, *et al.* Breast Cancer in Limited-Resource Countries: An Overview of the Breast Health Global Initiative 2005 Guidelines. *Breast J* 2006;**12**:S3–15. doi:10.1111/j.1075-122x.2006.00199.x

12 Anderson BO. Breast healthcare and cancer control in limited-resource countries: a framework for change. *Nat Clin Pract Oncol* 2006;**3**:4–5. doi:10.1038/ncponc0397

For peer review only

13 Anderson BO, Yip C-H, Ramsey SD, *et al.* Breast Cancer in Limited-Resource Countries: Health Care Systems and Public Policy. *Breast J* 2006;**12**:S54–69. doi:10.1111/j.1075-122x.2006.00203.x

14 Carlson RW, Scavone JL, Koh W-J, *et al.* NCCN Framework for Resource Stratification: A Framework for Providing and Improving Global Quality Oncology Care. *J National Compr Cancer Netw Jnccn* 2016;**14**:961–9. doi:10.6004/jnccn.2016.0103

15 Network NCC. NCCN Framework for Resource Stratification of NCCN Guidelines (NCCN Framework^{TM}). 2020.https://www.nccn.org/framework/ (accessed 29 Dec 2020).

16 Al-Sukhun S, Temin S, Chavez-MacGregor M, *et al.* ASCO Resource-Stratified Guidelines: Methods and Opportunities. *J Global Oncol* 2018;**4**:1–8. doi:10.1200/jgo.18.00113

17 Ku G, Tan IB, Yau T, *et al.* Management of colon cancer: resource-stratified guidelines from the Asian Oncology Summit 2012. *Lancet Oncol* 2012;**13**:e470-81. doi:10.1016/s1470-2045(12)70424-2

18 Peters MD, Godfrey CM, Khalil H, *et al.* Guidance for conducting systematic scoping reviews. *Int J Evid Based Healthc* 2015;**13**:141–6. doi:10.1097/xeb.0000000000000050

Participants	Guidelines developed for low-and middle-income countries across all fields of medicine
Concept	Development, implementation, and validation of resource-stratfied guidelines
Context	Limited to guidelines developed for low-and middle-income countries, as defined by world bank data for medical specialties recognized by the European Union and European Economic Area

Table 1. PCC inclusion criteria

For peer review only

Appendices

Appendix I: Search strategy

PubMed Search	Query	Records Retrieved
#1	(resource and(stratified or limited or limitations or stratification or low or constrain or constrained or under or driven or responsible or scarce or targeted or allocate* or management or specific or modified or adapt or adapted or adaptation*))	448,459
#2	("DEVELOPING COUNTRIES"[MH] OR "developing nation" OR "developing country" or "developing countries" OR((LMIC OR LMICS OR LICs OR MICs) OR "DEVELOPING COUNTRIES/ECONOMICS"[MH] OR("Low and middle-income country" OR "lower middle income country" or "Low-and middle-income countries" OR "lower middle-income countries") OR "low-income countries" or "low income country" OR "resource-poor setting" or "transitional country" or "transitional countries"))	175,829
#3	("CLINICAL PRACTICE GUIDELINE" OR "EVIDENCE-BASED RECOMMENDATIONS" OR(GUIDELINE OR GUIDELINES) OR(PROTOCOL) OR "EVIDENCE-BASED MEDICINE"[MH] OR "PRACTICE GUIDELINES AS TOPIC"[MH] or "Health Plan Implementation"[MAJR] OR "GUIDELINE ADHERENCE"[MH] OR "PRACTICE GUIDELINES AS TOPIC"[MH])	1,227,106
#4	#1 AND #2 AND #3	2,796
Filters	Human species; English Language	2,057

Scopus Search	Query	Records Retrieved
#1	TITLE-ABS-KEY (resource* W/3 (stratified OR limited OR limitations OR stratification OR low OR constrain* OR under OR driven OR responsible OR scarce OR targeted OR allocate* OR management OR specific OR modified OR adapt*))	381,768
#2	TITLE-ABS-KEY (("developing countr*" OR "developing nation" OR (lmic* OR lics OR mics) OR "Low* and middle-income countr*" OR ("low-income countr*" OR "resource-poor setting" OR "transitional countr*")))	349,488
#3	TITLE-ABS-KEY ("Clinical Practice Guideline*" OR "Evidence-Based Recommendation*" OR guideline* OR protocol OR "Evidence-Based Medicine" OR "Health Plan Implementation" OR "Guideline Adherence")	2,240,877
#4	#1 AND #2 AND #3	2,168
Filters	Human species; English Language	1,703

Search conducted 4 Nov 2021

Appendix II: Data extraction instrument

Title	Author	Population Characteristics of Developers	Intended Country for Implementation	Rationale for Development	Methodology of Development	Development	Implementation	Validation	Summary of finding	Medical speciality	Practice implications	Recommendations for further development

BMJ Open

Development, Implementation, and Validation of Resource Stratified Guidelines in Low-and Middle-Income Countries: A Scoping Review Protocol

Journal:	*BMJ Open*
Manuscript ID	bmjopen-2021-059603.R2
Article Type:	Protocol
Date Submitted by the Author:	26-Jun-2022
Complete List of Authors:	Griswold, Dylan; Cambridge University, Clinical Neurosciences<br>Venturini, Sara; University of Cambridge, NIHR Global Health Research Group on Neurotrauma; University of Cambridge, Department of Clinical Neurosciences<br>Carney, Nancy; Oregon Health & Science University, Department of Medical Informatics and Clinical Epidemiology<br>Rubiano, Andres M; Universidad El Bosque, Neurosciences Institute; University of Cambridge, NIHR Global Health Research Group on Neurotrauma<br>Hutchinson, Peter; University of Cambridge, NIHR Global Health Research Group on Neurotrauma; University of Cambridge, Department of Clinical Neurosciences<br>Kolias, Angelos; Addenbrooke's Hospital, NIHR Global Health Research Group on Neurotrauma; University of Cambridge, Department of Clinical Neurosciences
**Primary Subject Heading**:	Evidence based practice
Secondary Subject Heading:	Global health, Research methods
Keywords:	Protocols & guidelines < HEALTH SERVICES ADMINISTRATION & MANAGEMENT, Quality in health care < HEALTH SERVICES ADMINISTRATION & MANAGEMENT, International health services < HEALTH SERVICES ADMINISTRATION & MANAGEMENT

SCHOLARONE™ Manuscripts

Development, Implementation, and Validation of Resource Stratified Guidelines in Low-and Middle-Income Countries: A Scoping Review Protocol

Dylan P Griswold[1,2,3] Sara Venturini[1,2] Nancy A. Carney[4] Andres M. Rubiano[1,5,6] Peter J. Hutchinson[1,2]Angelos G. Kolias[1,2]

[1] NIHR Global Health Research Group on Neurotrauma, University of Cambridge, Cambridge, UK
[2] Division of Neurosurgery, Department of Clinical Neurosciences, Addenbrooke's Hospital & University of Cambridge, Cambridge, UK [3] Stanford University School of Medicine, Stanford, CA, USA [4] Department of Medical Informatics and Clinical Epidemiology, School of Medicine, Oregon Health & Science University, Portland, Oregon, USA [5] Neuroscience Institute, INUB-MEDITECH Research Group, El Bosque University, Bogotá, Colombia [6] Neurological Surgery Service, Vallesalud Clinic, Cali, Colombia

Keywords: scoping review; developing countries; evidence-based guidelines; resource stratified guidelines; LMICs; low-and middle-income countries; global health

Word count: 1814

Correspondence:

Dylan Griswold

Division of Neurosurgery

Department of Clinical Neurosciences

Addenbrooke's Hospital

University of Cambridge

Cambridge CB2 0QQ

dylgris@gmail.com

ABSTRACT

Introduction: Nearly every field of medicine has some form of clinical practice guidelines. However, only within the past five to ten years has the medical community acknowledged the need for well-developed guidelines tailored to the local healthcare needs and the resources available. In most low-and middle-income countries (LMICs) healthcare workers depend on guidelines developed in high-income countries (HICs), yet many interventions validated in a HIC are ineffective when implemented in an LMIC. The variation in infrastructure, medical personnel, technology, and environmental conditions exhibited in LMICs relative to HICs necessitates a careful appraisal of the evidence base used in clinical guideline recommendations. This review aims to map the use of resource-stratified guidelines across all fields of medicine. The review seeks to answer three questions for the identified guidelines, 1) what was the method of development, 2) have they been implemented, and if so, 3) have they been validated.

Methods

The search strategy will aim to locate studies from inception to November 2021. An initial limited search of PubMed and Scopus was undertaken to identify articles on the topic. The text words contained in the titles and abstracts of relevant articles, and the index terms used to describe the articles were used to develop a full search strategy for PubMed and Scopus. This scoping review will be conducted in accordance with the JBI methodology for scoping reviews. Data to be extracted from each study will include population characteristics of both developers and intended implementation population, medical specialty, validation status, method of guideline development, whether the study is consensus or evidence-based in addition to a summary of recommendations for practice.

Ethics and dissemination Ethical approval is not required for this review. The plan for dissemination is to publish review findings in a peer-reviewed journal.

For peer review only

ARTICLE SUMMARY

Strengths and Limitations of this study

- To the best of our knowledge this protocol provides a detailed description of the first scoping review on the development, implementation, and validation of resource-stratified guidelines in low-and middle-income countries.
- The protocol adheres to the rigorous JBI methodology for scoping reviews
- The protocol is being conducted by a multidisciplinary team with extensive experience of conducting high-quality reviews
- Given the narrow specificity of the review, very few papers may be included in the final analysis

For peer review only

INTRODUCTION

Best-practice, evidence-based, clinical practice guidelines have become the gold standard for patient care. However, these guidelines are, by and large, developed by High-Income Countries (HICs). Thus, data extracted from clinical trials and adequate statistically controlled comparative studies used to develop the guidelines are based on the enrolment population. Of the 173,532 trials registered on the International Clinical Trials Registry Platform (ICTRP) between 2005 to 2013, 85% were registered to recruit in HICs.[1] For the same period, 4.7% were registered to recruit in lower-middle-income countries (LMICs) and 0.8% in low-income countries (LICs). Thus, 85% of those studies registered for recruitment in high-income countries have a very different sample population relative to the same study carried out with a sample population enrolled from LMICs with social, cultural, genetic, and infrastructure differences.[1] This difference is real and has implications for implementing guidelines developed for and by HICs in an LMIC. In most LMICs healthcare workers depend on guidelines developed in HICs, yet many interventions validated in a HIC are ineffective when implemented in an LMIC.[2,3] The variation in infrastructure, medical personnel, technology, and environmental conditions exhibited in LMICs relative to HICs necessitates a careful appraisal of the evidence base used in clinical guideline recommendations.

Nearly every field of medicine has some form of clinical practice guidelines.[4] Nevertheless, only within the last 5-10 years has the literature acknowledged the need for well-developed guidelines tailored to the local healthcare needs and the resources available. The WHO has developed guidelines for different topics ranging from antimicrobial resistance and neglected tropical disease to environmental health and health systems.[5] Dr. Charles Mock et al. were renegades in this regard, publishing Trauma Mortality Patterns in Three Nations at Different Economic Levels: Implications for Global Trauma System Development – in 1998.[6] The outcome of the study showed that in all three cities, the majority of deaths occurred in the prehospital setting and that improving emergency care is especially vital in middle-income nations that have already established essential emergency medical services (EMS). Mock then led an initiative between the International Association for the Surgery of Trauma and Surgical Intensive Care (IATSIC) and the WHO to establish the Working Group for Essential Trauma Care. The group sought to formulate a plan to address the difficulties that confront trauma care in LMICs. In 2004, the group published the document, Guidelines for essential trauma care, which set forth a list of essential trauma services in 14 categories of trauma care that the group felt was achievable in nearly every worldwide setting and included the human and physical resources that would ensure their provision.[7]

Though guidelines like those developed by Mock's team are available, they are situational, for which there are no comprehensive guidelines for each of the 14 categories of trauma care.

WHO's current trauma guidelines recognize that some protocols developed by HICs require the most costly resources in the "modern therapeutic armamentarium," and that, "it is unlikely that low- or even middle-income countries will be able to meet these guidelines fully."[7] Thus, the WHO's guidelines list the most essential diagnostic and therapeutic capabilities from HIC trauma guidelines. These recommendations outline essential procedures that the vast majority of LMIC facilities can perform. However, LMICs cannot implement these as official trauma guidelines as they fail to provide recommendations stratified by the resources available across multiple domains and levels of care for severe trauma.

One model that has demonstrated success is the resource-stratified guidelines developed for oncology care in areas with varying levels of resource availability. The Breast Health Global Initiative (BHGI) was organized in 2002 to improve the care of women with breast cancer in low-resource settings.[8] Their initiative was successful in developing clinical practice guidelines that account for varying levels of resources available in differing regions. Their methodology provides a framework for diagnosis and treatment recommendations across four resource levels: basic, limited, enhanced, and maximal.[9–13] In recent years the National Comprehensive Cancer Network (NCCN) have taken their evidence-based, consensus-driven clinical practice guidelines (NCCN Guidelines) developed for high-resouce settings, and have developed a framework for resource-stratifying the guidelines for implementation in resource-limited settings. They have formally designated this framework as the NCCN Framework for Resource Stratification of the NCCN Guidelines (NCCN Framework).[14] Like the BHGI guidelines, the NCCN Framework also identifies four resource environments: basic resources, core resources, enhanced resources, and NCCN Guideline assumed resources. The current library of guidelines includes 16 different types of cancers in addition to breast cancer screening, adult cancer pain, and palliative care.[15]

For peer review only - http://bmjopen.bmj.com/site/about/guidelines.xhtml

According to 2016 data, 47% of the 754,000 verified users of the internet-based guidelines were from 198 countries outside the United States.[14]

While the NCCN Framework, has led to the development of a variety of cancer-specific resource-stratified guidelines, they have yet to be translated across other fields of medicine. The American Society of Clinical Oncology (ASCO) and the Asian Oncology Society have also developed their own set of cancer-specific resource-stratified guidelines.[16,17] It is largely unknown if and to what degree other fields of medicine have similar frameworks. Understanding how other fields of medicine have developed and implemented resource-stratified guidelines can inform development of new resource-stratified guidelines for specialities without them. Therefore, this review aims to map the use of resource-stratified guidelines across all fields of medicine. A preliminary search of PROSPERO, MEDLINE, the Cochrane Database of Systematic Reviews, and the JBI Database of Systematic Reviews and Implementation Reports was conducted, and no current or underway systematic reviews on the topic were identified.

The findings of this review will inform stakeholders about relevant models to develop, implement, and evaluate resource-stratified- guidelines in low-resource settings.

METHODS

The proposed scoping review will be conducted in accordance with the JBI methodology for scoping reviews.[18]

Review question

Question: Of the resource-stratified guidelines being used in medicine, what are the methods of development, have they been successfully implemented, and if so, have they been validated? Validation refers to whether the performance of the guidelines was assessed, and if so, the results from the assessment.

Objective: To identify resource-stratified guidelines being used in medicine and assess their methods of development, implementation, and validation.

Patient and public involvement

Patients and the public were not involved in the design of this scoping review protocol.

Data source and search strategy

The search strategy will aim to locate both published and unpublished studies from inception to November 2021. An initial limited search of PubMed and Scopus was undertaken to identify articles on the topic. The text words contained in the titles and abstracts of relevant articles, and the index terms used to describe the articles were used to develop a full search strategy for PubMed and Scopus. A full search strategy for PubMed and Scopus is detailed in Appendix I. In the second phase of the search, a final search strategy will be adopted for each information source. The reference lists of all selected studies will be screened for additional studies during the third phase of the search. Unpublished studies will be captured through scanning societal meetings for abstracts and consensus-based guideline development conferences.

Selection of studies

Following the search, all identified citations will be collated and uploaded into EndNoteX9 (Clarivate Analytics, PA, USA). The citations will then be imported into Covidence online software (Veritas Health Innovation, Melbourne, Australia) for screening. Two independent researchers will examine titles and abstracts for inclusion. The full text of selected studies will be retrieved and assessed. Full-text studies that do not meet the inclusion criteria will be excluded, and the reasons for exclusion will be provided in an appendix in the final scoping review. Any disagreements that arise between the researchers during either title and abstract screening or full-text screening will be resolved through discussion, or with a third reviewer. The results of the search will be reported in full in the final article and presented in a Preferred Reporting Items for Systematic Reviews and Meta-Analyses extension for Scoping Reviews (PRISMA-ScR) checklist.

For peer review only

For peer review only - http://bmjopen.bmj.com/site/about/guidelines.xhtml

Eligibility criteria

Inclusion criteria

Participants

This scoping review will consider guidelines that were developed for low-and middle-income countries, as defined by world bank data for medical specialties recognized by the European Union and European Economic Area.

Concept

The concept of interest for the proposed scoping review is resource-stratified guidelines developed for low-and middle-income countries across all fields of medicine. This will include, but not be limited to, population, medical specialty, intervention, validation status, method of guideline development, whether the study is consensus or evidence-based in addition to the comparator and outcome measures.

Context

The review will be limited to guidelines developed for low-and middle-income countries, as defined by world bank data for medical specialties recognized by the European Union and European Economic Area.

Types of studies

This scoping review will consider both experimental and quasi-experimental study designs including randomized controlled trials, non-randomized controlled trials, before and after studies, and interrupted time-series studies. In addition, analytical observational studies, including prospective and retrospective cohort studies, case-control studies, and analytical cross-sectional studies, will be considered for inclusion.

Studies published in English and English abstracts of foreign language studies will be included. All studies will be considered regardless of publication date.

The PCC inclusion criteria is summarized in Table 1.

Data extraction

Data will be extracted from the papers included in the review by two independent researchers using the data extraction instrument (Appendix II). The following information will be extracted from the articles: (1) study title; (2) author; (3) population characteristics of guideline developers; (4) intended population for implementation; (5) rationale for development; (6) methodology (7) development; (8) implementation; (9) validation; (10) summary of findings; (11) medical speciality; 12) practice implications; (13) recommendations for further development

The draft data extraction tool will be modified and revised as necessary during the process of extracting data from each included study. Modifications will be detailed in the full scoping review report. Any disagreements that arise between the reviewers will be resolved through discussion, or with a third reviewer. Authors of papers will be contacted to request missing or additional data, where required.

Data presentation

The extracted data will be presented in tabular form and as a narrative summary that aligns with the aim of this scoping review. The table will report: i) distribution of studies by countries of guideline origin and intended population; ii) methodology of development; iii) implementation and validation; iv); summary of recommendations. Graphical representations may be used, including bar charts, line charts, pie charts, and diagrams.

For peer review only

Ethics and dissemination

No ethical approval will be required, as this review is based on already published data and does not involve interaction with human subjects. The plan for dissemination, however, is to publish the findings of the review in a peer-reviewed journal and present findings at high- level international conferences that engage the most pertinent stakeholders.

DISCUSSION

This protocol has been rigorously developed and designed specifically to determine the methods of development, implementation, and validation of resource-stratified guidelines being used across all fields of medicine. The findings of this review will inform stakeholders about relevant models to develop, implement, and evaluate resource-stratified guidelines in low-resource settings.

Limitations

This study will be limited by the relative novelty of guideline development for LMICs. Based upon our initial search there are very few examples of resource-stratified guidelines. However, the purpose of the review is to identify those that do exist and to extract the elements that will aid in future development of resource-stratified guidelines in LMICs.

For peer review only

For peer review only - http://bmjopen.bmj.com/site/about/guidelines.xhtml

Contributors DG, AK, PJH, AMR conceived the review. NC and SV helped with the development of the methods. DG drafted the manuscript and all authors contributed to its revision.

Funding This work is supported by the NIHR Global Health Research Group on Neurotrauma, which was commissioned by the National Institute for Health Research (NIHR) using UK aid from the UK Government (project 16/137/105). The views expressed in this publication are those of the author(s) and not necessarily those of the NIHR or the Department of Health and Social Care. D.P.G. is supported by the Gates Cambridge Trust (OPP1144). P.J.H. is supported by a Research Professorship from the NIHR, the NIHR Cambridge Biomedical Research Centre, a European Union Seventh Framework Program grant (CENTER-TBI; grant no. 602,150), and the Royal College of Surgeons of England. A.G.K. is supported by a Clinical Lectureship, School of Clinical Medicine, University of Cambridge and the Royal College of Surgeons of England.

Competing interests None declared.

Patient consent for publication Not required.

Data statement: This review is based on previously published data. Any relevant data will be published with the review as either an appendix or as an online supplement.

Acknowledgements The authors would like to thank Isla Kuhn, Head of Medical Library Services, University of Cambridge Medical Library for her assistance in developing the search strategy

Open access This is an open access article distributed in accordance with the Creative Commons Attribution Non Commercial (CC BY-NC 4.0) license, which permits others to distribute, remix, adapt, build upon this work non-commercially, and license their derivative works on different terms, provided the original work is properly cited, appropriate credit is given, any changes made indicated, and the use is non-commercial. See: http://creativecommons.org/licenses/by-nc/4.0/.

License to BMJ Group for Publication: I *Dylan Griswold,* the Corresponding Author of this article contained within the original manuscript which includes any diagrams & photographs within and any related or stand-alone film submitted (the Contribution") has the right to grant on behalf of all authors and does grant on behalf of all authors, a licence to the BMJ Publishing Group Ltd and its licencees, to permit this Contribution (if accepted) to be published in the BMJ and any other BMJ Group products and to exploit all subsidiary rights, as set out in our licence set out at: http://www.bmj.com/about-bmj/resources-authors/forms-policies-and-checklists/copyright-open-access-and-permission-reuse.

For peer review only

1 Viergever RF, Li K. Trends in global clinical trial registration: an analysis of numbers of registered clinical trials in different parts of the world from 2004 to 2013. *Bmj Open* 2015;**5**:e008932. doi:10.1136/bmjopen-2015-008932

2 Buekens P, Keusch G, Belizan J, *et al.* Evidence-Based Global Health. *Jama* 2004;**291**:2639–41. doi:10.1001/jama.291.21.2639

3 Olayemi E, Asare EV, Kuma AAB. Guidelines in lower-middle income countries. *Brit J Haematol* 2017;**177**:846–54. doi:10.1111/bjh.14583

4 WEISZ G, CAMBROSIO A, KEATING P, *et al.* The Emergence of Clinical Practice Guidelines: The Emergence of Clinical Practice Guidelines. *Milbank Q* 2007;**85**:691–727. doi:10.1111/j.1468-0009.2007.00505.x

5 Organization"] ["World Health. WHO guidelines approved by the Guidelines Review Committee. 2020.

6 Mock CN, Jurkovich GJ, nii-Amon-Kotei D, *et al.* Trauma Mortality Patterns in Three Nations at Different Economic Levels: Implications for Global Trauma System Development. *J Trauma Inj Infect Critical Care* 1998;**44**:804–14. doi:10.1097/00005373-199805000-00011

7 Mock C, Organization) Essential Trauma Care Project (World Health, Organization. World Health, *et al. Guidelines for essential trauma care*. Geneva: : World Health Organization 2004.

8 Anderson BO, Braun S, Carlson RW, *et al.* Overview of Breast Health Care Guidelines for Countries with Limited Resources. *Breast J* 2003;**9**:S42–50. doi:10.1046/j.1524-4741.9.s2.3.x

9 Anderson BO, Carlson RW. Guidelines for Improving Breast Health Care in Limited Resource Countries: The Breast Health Global Initiative. *J Natl Compr Canc Ne* 2007;**5**:348–55. doi:10.6004/jnccn.2007.0031

10 Anderson BO, Yip C, Smith RA, *et al.* Guideline implementation for breast healthcare in low-income and middle-income countries. *Cancer* 2008;**113**:2221–43. doi:10.1002/cncr.23844

11 Anderson BO, Shyyan R, Eniu A, *et al.* Breast Cancer in Limited-Resource Countries: An Overview of the Breast Health Global Initiative 2005 Guidelines. *Breast J* 2006;**12**:S3–15. doi:10.1111/j.1075-122x.2006.00199.x

12 Anderson BO. Breast healthcare and cancer control in limited-resource countries: a framework for change. *Nat Clin Pract Oncol* 2006;**3**:4–5. doi:10.1038/ncponc0397

For peer review only

13 Anderson BO, Yip C-H, Ramsey SD, *et al.* Breast Cancer in Limited-Resource Countries: Health Care Systems and Public Policy. *Breast J* 2006;**12**:S54–69. doi:10.1111/j.1075-122x.2006.00203.x

14 Carlson RW, Scavone JL, Koh W-J, *et al.* NCCN Framework for Resource Stratification: A Framework for Providing and Improving Global Quality Oncology Care. *J National Compr Cancer Netw Jnccn* 2016;**14**:961–9. doi:10.6004/jnccn.2016.0103

15 Network NCC. NCCN Framework for Resource Stratification of NCCN Guidelines (NCCN Framework^{TM}). 2020.https://www.nccn.org/framework/ (accessed 29 Dec 2020).

16 Al-Sukhun S, Temin S, Chavez-MacGregor M, *et al.* ASCO Resource-Stratified Guidelines: Methods and Opportunities. *J Global Oncol* 2018;**4**:1–8. doi:10.1200/jgo.18.00113

17 Ku G, Tan IB, Yau T, *et al.* Management of colon cancer: resource-stratified guidelines from the Asian Oncology Summit 2012. *Lancet Oncol* 2012;**13**:e470-81. doi:10.1016/s1470-2045(12)70424-2

18 Peters MD, Godfrey CM, Khalil H, *et al.* Guidance for conducting systematic scoping reviews. *Int J Evid Based Healthc* 2015;**13**:141–6. doi:10.1097/xeb.0000000000000050

Participants	Guidelines developed for low-and middle-income countries across all fields of medicine
Concept	Development, implementation, and validation of resource-stratfied guidelines
Context	Limited to guidelines developed for low-and middle-income countries, as defined by world bank data for medical specialties recognized by the European Union and European Economic Area

Table 1. PCC inclusion criteria

For peer review only

Appendices

Appendix I: Search strategy

PubMed Search	Query	Records Retrieved
#1	(resource and(stratified or limited or limitations or stratification or low or constrain or constrained or under or driven or responsible or scarce or targeted or allocate* or management or specific or modified or adapt or adapted or adaptation*))	448,459
#2	("DEVELOPING COUNTRIES"[MH] OR "developing nation" OR "developing country" or "developing countries" OR((LMIC OR LMICS OR LICs OR MICs) OR "DEVELOPING COUNTRIES/ECONOMICS"[MH] OR("Low and middle-income country" OR "lower middle income country" or "Low-and middle-income countries" OR "lower middle-income countries") OR "low-income countries" or "low income country" OR "resource-poor setting" or "transitional country" or "transitional countries"))	175,829
#3	("CLINICAL PRACTICE GUIDELINE" OR "EVIDENCE-BASED RECOMMENDATIONS" OR(GUIDELINE OR GUIDELINES) OR(PROTOCOL) OR "EVIDENCE-BASED MEDICINE"[MH] OR "PRACTICE GUIDELINES AS TOPIC"[MH] or "Health Plan Implementation"[MAJR] OR "GUIDELINE ADHERENCE"[MH] OR "PRACTICE GUIDELINES AS TOPIC"[MH])	1,227,106
#4	#1 AND #2 AND #3	2,796
Filters	Human species; English Language	2,057

For peer review only - http://bmjopen.bmj.com/site/about/guidelines.xhtml

Scopus Search	Query	Records Retrieved
#1	TITLE-ABS-KEY (resource* W/3 (stratified OR limited OR limitations OR stratification OR low OR constrain* OR under OR driven OR responsible OR scarce OR targeted OR allocate* OR management OR specific OR modified OR adapt*))	381,768
#2	TITLE-ABS-KEY (("developing countr*" OR "developing nation" OR (lmic* OR lics OR mics) OR "Low* and middle-income countr*" OR ("low-income countr*" OR "resource-poor setting" OR "transitional countr*")))	349,488
#3	TITLE-ABS-KEY ("Clinical Practice Guideline*" OR "Evidence-Based Recommendation*" OR guideline* OR protocol OR "Evidence-Based Medicine" OR "Health Plan Implementation" OR "Guideline Adherence")	2,240,877
#4	#1 AND #2 AND #3	2,168
Filters	Human species; English Language	1,703

Search conducted 4 Nov 2021

Appendix II: Data extraction instrument

Title	Author	Population Characteristics of Developers	Intended Country for Implementation	Rationale for Development	Methodology of Development	Development	Implementation	Validation	Summary of finding	Medical speciality	Practice implications	Recommendations for further development

For peer review only
